# The biosurfactant viscosin transiently stimulates n-hexadecane mineralization by a bacterial consortium

**DOI:** 10.1007/s00253-014-6054-3

**Published:** 2014-09-14

**Authors:** Frederik Bak, Lise Bonnichsen, Niels O. G. Jørgensen, Mette H. Nicolaisen, Ole Nybroe

**Affiliations:** Section of Genetics and Microbiology, Department of Plant and Environmental Sciences, University of Copenhagen, Thorvaldsensvej 40, 1871 Frederiksberg, Copenhagen Denmark

**Keywords:** Alkanes, Biodegradation, Lipopeptides, *Pseudomonas*

## Abstract

*Pseudomonas* produces powerful lipopeptide biosurfactants including viscosin, massetolide A, putisolvin, and amphisin, but their ability to stimulate alkane mineralization and their utility for bioremediation have received limited attention. The four *Pseudomonas* lipopeptides yielded emulsification indices on hexadecane of 20–31 % at 90 mg/l, which is comparable to values for the synthetic surfactant Tween 80. Viscosin was the optimal emulsifier and significantly stimulated n-hexadecane mineralization by diesel-degrading bacterial consortia but exclusively during the first 2 days of batch culture experiments. Growth of the consortia, as determined by OD_600_ measurements and quantification of the *alkB* marker gene for alkane degradation, was arrested after the first day of the experiment. In contrast, the control consortia continued to grow and reached higher OD_600_ values and higher *alkB* copy numbers during the next days. Due to the short-lived stimulation of n-hexadecane mineralization, the stability of viscosin was analyzed, and it was observed that added viscosin was degraded by the bacterial consortium during the first 2 days. Hence, viscosin has a potential as stimulator of alkane degradation, but its utility in bioremediation may be limited by its rapid degradation and growth-inhibiting properties.

## Introduction

Pollution with petroleum hydrocarbons is widespread and represents a problem for the environment as well as human health. Petrol and diesel contain a high amount of aliphatic hydrocarbons, in particular C_14_ to C_20_ alkanes (Liang et al. 2005; Stroud et al. 2007). The potential to degrade alkanes has been identified in a great variety of bacteria including *Proteobacteria* as well as *Actinomycetales* (van Beilen and Funhoff 2007). The enzyme alkane hydroxylase, AlkB, is responsible for the first step in degradation of C_5_–C_16_ alkanes (Ji et al. 2013), and the *alkB* gene has been widely used as a marker for the microbial alkane degradation potential due to its widespread occurrence among degraders (Perez-de-Mora et al*.* 2011; Jurelevicius et al. 2013).

Microbial degradation of alkanes is often limited by the low bioavailability of these hydrophobic pollutants. Microorganisms employ two general strategies to access hydrophobic substrates and hence circumvent the limiting bioavailability. First, they can rely on biosurfactant-mediated solubilization. Biosurfactants are amphipathic molecules with varying structures produced by a diverse group of bacteria (Van Hamme et al. 2006). They can facilitate interactions between the bacteria and alkanes by generating emulsions or by pseudosolubilization of the alkane into hydrophilic micelles (Perfumo et al. 2010; Ward 2010). Second, bacteria can access alkane drops by direct cell contact and even form biofilms on the hydrophobic substrate (Perfumo et al. 2010). Biosurfactants also play a role in the direct access strategy by increasing the cell surface hydrophobicity of the degrading organism, thereby increasing adherence to the hydrophobic substrate (Hommel 1990; Bouchez-Naitali et al. 1999; Zhang and Miller 1995; Kumar et al. 2008; Al-Tahhan et al. 2000; Perfumo et al. 2010; Ward 2010; Djeridi et al. 2013). Biosurfactants may even affect the direct access strategy in a more indirect manner, as they play several roles in biofilm formation, and in some cases have been shown to dissolve established biofilms (Tribelli et al. 2012; Whyte et al. 1999; Ward 2010; Rivardo et al. 2009; Kuiper et al. 2004)

Various studies have been conducted on addition of biosurfactants such as rhamnolipids (Lawniczak et al. 2013) and the *Bacillus* lipopeptide surfactin (Moran et al. 2000; Olivera et al. 2000; Whang et al*.* 2008) in bioremediation processes of petroleum hydrocarbon/alkane pollutions. Often, biodegradation is stimulated by these biosurfactants (Moran et al. 2000; Kang et al. 2010), but inhibitory effects have also been observed and may be caused by toxicity of the biosurfactant, by formation of biosurfactant micelles that offer limited access to the substrate or by destruction of degrading biofilms (Van Hamme and Ward 2001; Zeng et al. 2011; Chrzanowski et al. 2011; Kuiper et al. 2004). The use of biosurfactants in bioremediation is however still hampered by lack of detailed understanding of the impact of biosurfactants on degrader activity and performance in natural environments.

It is often highlighted that biosurfactants are biodegraded easier than chemical surfactants (Mulligan 2005), but fast biodegradation of an exogenously added biosurfactant may not only be advantageous, as fast degradation may limit its potential for usage in bioremediation. However, the stability of biosurfactants is rarely investigated in alkane biodegradation studies (Frank et al. 2010).

Most studies of biosurfactant-facilitated alkane degradation have focused on single strains or mixtures of a few strains. These studies have improved understanding of the detailed mechanisms of biosurfactant-mediated hydrocarbon uptake but even revealed that such mechanisms are often strain-specific (Thavasi 2011; Ward 2010). Thus, the effects of biosurfactants added to degrader consortia are still lacking attention to improve realism of the obtained conclusions. Bacteria in a consortium probably use different hydrocarbon accession strategies, and Owsianiak et al. (2009) suggested that addition of biosurfactants to consortia stimulates biodegradation performed by the degraders taking up solubilized hydrocarbons. On the other hand, bacteria using the direct substrate accession mode may be negatively affected by biosurfactants.

*Pseudomonas* produces several powerful lipopeptide biosurfactants with low critical micelle concentrations (CMCs) (Raaijmakers et al. 2010). These lipopeptides, including the compounds viscosin, massetolide A, putisolvin, and amphisin, have primarily been investigated for their antimicrobial properties and their effects on biofilm formation (Raaijmakers et al. 2010). However, the ability of *Pseudomonas* lipopeptides to stimulate mineralization of hydrophobic hydrocarbons and their utility for bioremediation have so far received very limited attention. The aims of the current study are (1) to provide information on the ability of *Pseudomonas* lipopeptides to solubilize the model alkane, n-hexadecane; (2) to determine the impact of the lipopeptide viscosin on mineralization of n-hexadecane and growth by a bacterial consortium; and (3) to obtain information about the stability of viscosin in the presence of the n-hexadecane-degrading consortium.

## Materials and methods

### Isolation of a diesel-degrading bacterial consortium from soil

Diesel-degrading bacteria were isolated from soils polluted with hydrocarbons (Aabenraa, Denmark). Initially, bacteria were extracted from the soils as previous described by Johnsen and Karlson (2005) with a few adjustments. Suspensions of 0.50 g soil and 4.5-ml 2 mM sodium pyrophosphate solution (Na_4_P_2_O_7_·10H_2_O) (pH = 8.0) were mixed for 30 min on a gyratory shaker. The suspensions were streaked on Bushnell Haas (BH) (Sigma-Aldrich) agar plates with 0.1 % sterile filtered diesel (Shell) and 0.1 % nystatin (ICN Biomedicals Inc.) to inhibit fungal growth. Plates were incubated for 2 weeks at room temperature. Colony material from the plates was washed off by 0.9 % NaCl and used as inoculum for a culture in liquid BH medium with 0.1 % sterile filtered diesel and 0.1 % nystatin. The culture was incubated for 13 days at room temperature with stirring, whereupon the culture (diesel-degrading consortium) was stored at −70 °C as 1-ml aliquots of a 50 % *v*/*v* glycerol/bacterial suspension.

### Purification of lipopeptide biosurfactants

The lipopeptides viscosin, massetolide A, putisolvin, and amphisin were purified from the producing strains *Pseudomonas fluorescens* SBW25 (Thomson et al. 1995), *P. fluorescens* SS101 (de Souza et al. 2003), *Pseudomonas putida* 267 (Tran et al. 2008) and *Pseudomonas* sp. DSS73 (Sørensen et al. 2001), respectively, essentially as described by de Souza et al. (2003). Briefly, the strains were cultivated on King’s B agar plates in darkness at 28 °C for 1 day, before being transferred to 20 °C and incubated for another 3 days. Colony material was suspended in demineralized water (Milli-Q, Millipore; referred to as Milli-Q water hereafter) and homogenized by shaking. Cells and supernatant was separated two times by centrifugation at 4,700 rpm for 20 min at 4 °C on a Sigma 3-18K centrifuge (Sciquip). The supernatant was acidified to pH 2.0 with 1 M HCl and left overnight on ice for a precipitate to form. The solution including precipitate was centrifuged in sterile centrifuge tubes for 27 min at 7,000 rpm and 4 °C in a Sigma 3-18K centrifuge. The supernatant was discarded, and the precipitate was washed four times with Milli-Q water at pH 2.0. The precipitate was dissolved in Milli-Q water, and pH was adjusted to 8.0 with 1 M NaOH to fully dissolve the precipitate. The solution was lyophilized, and the purity of the lipopeptide preparations was qualitatively analyzed by high-performance liquid chromatography.

### High-performance liquid chromatography

High-performance liquid chromatography (HPLC) analysis was carried out using a Waters Alliance series 2695 system and a Waters model 996 photodiode array detector (www.waters.com). Chromatographic analysis of the lipopeptides followed the protocol by Nielsen and Sørensen (2003) with minor modifications. Briefly, a Hypersil BDS C_18_ column (100 × 4.6 mm and 3 μm particle size) (www.thermoscientific.com) was used for separation of the lipopeptides. Solvents were HPLC-grade acetonitrile (solvent A) and 0.1 % *o*-phosphoric acid (solvent B), mixed in a linear gradient of 15 to 100 % solvent A from 0 to 40 min, and of 100 to 15 % solvent A between 40 and 44 min. The flow rate was 1 ml/min, and the column temperature was 40 °C. The injected sample volume was 10 μl. The lipopeptides were monitored at wavelengths of 190 to 250 nm. For quantification of viscosin in cultures of the diesel degrading consortium, a wavelength of 220 nm was applied and viscosin concentrations were calculated using purified viscosin as a standard. Handling of chromatographic data was performed using Waters Empower 2 software.

### Emulsification of n-hexadecane by lipopeptide biosurfactants

The emulsification index of viscosin, amphisin, massetolide A, and putisolvin, as well as the synthetic surfactant Tween 80 (Applichem), was determined using the assay described by Cooper and Goldenberg (1987). The surfactants were dissolved in Milli-Q water (pH 7.3) at 20 and 90 mg/l. Solutions (5 ml) were aliquoted into disposable pyrex tubes and overlaid with 5 ml n-hexadecane (Sigma-Aldrich) before vortexing at high speed for 2 min. Tubes were left for 24 h at room temperature before measuring the height of the emulsified layer and the total height of the sample. The emulsification index (E_24_) was estimated as height of the emulsified layer / total height of sample × 100 %.

### n-Hexadecane mineralization and growth of the bacterial consortium

The effect of viscosin on n-hexadecane mineralization by the degrading consortium isolated in this study was analyzed by a ^14^C-n-hexadecane mineralization assay. Prior to mineralization experiments, the consortium was inoculated in BH medium with 0.1 % diesel at 28 °C on a shaker with 150 rpm. After 24 h, a 0.1-ml aliquot was used to establish a culture in BH medium with 0.1 % n-hexadecane, which was incubated under the same conditions and used as inoculum for the mineralization experiments.

The mineralization assays were carried out for 14 days in infusion flasks containing 20-ml BH medium with 100 ppm n-hexadecane (Sigma-Aldrich), 3.15 nCi [l-^14^C] hexadecane at a specific activity of 55 mCi/mmol (http://www.arc-inc.com/), and 100-ppm trace element solution (Kragelund and Nybroe 1994). Viscosin was added to a final concentration of 90 mg/l. Flasks were inoculated with the consortium preculture to obtain a starting concentration of approximately 10^7^ cells/ml. ^14^CO_2_ was collected in glass tubes with 1-M NaOH solutions, and radioactivity was measured on a Beckman LS 1801 liquid scintillation counter (Beckman Instruments, Inc.). Data were corrected for background activity (typical value of 30 DPM). Growth was determined as optical density at 600 nm (OD_600_) measured by an UV-mini 1240 spectrophotometer (Shimadzu). For HPLC analysis, samples of 0.5 ml were removed and stored at −20 °C until analysis was carried out.

### DNA extraction

Samples for DNA extraction (1 ml) were collected from above experiments in RNase/DNase-free tubes. Cell pellets were obtained by removing 900 μl of the supernatant, and the pellets were immediately flash frozen in liquid nitrogen and stored at −70 °C. Prior to extraction, 100 μl of lysozyme (1 mg/ml, Sigma-Aldrich) in Tris-EDTA buffer (Sigma-Aldrich) were added to the pellet and left for 20 min at room temperature. For DNA extraction, the AllPrep DNA/RNA mini kit (Qiagen, USA) was used according to the manufacturer’s protocol. The final DNA extractions were stored at −70 °C until further processing.

### Quantification of the *alkB* genes by qPCR

The copy numbers of *alkB* genes were quantified using qPCR. The qPCR was carried out using a MX3000P® qPCR system (Stratagene). The a*lkB* genes were amplified using primers *alkB*-f (5′-AAYACIGCICAYGARCTIGGICAYAA-3′) and *alkB*-r (5′-GCRTGRTGRTCIGARTGICGYTG-3′) (Perez-de-Mora et al. 2011). This primer set amplifies *alkB* genes from a range of Gram-positive as well as Gram-negative bacteria without an obvious specificity for any bacterial group, and the obtained PCR product has the length of approximately 550 bp. Amplification of the fragment was carried out in 20-μl reactions using the Brilliant Master mix SYBR® GREEN which included the following: PCR buffer, dNTPs, 5 mM MgCl_2_, Sure start Taq polymerase, and SYBR Green 1 dye (Agilent Technologies, Santa Clara, CA). Further, reactions contained 0.8 μl of each primer (10 pmol/μl), 2 μl bovine serum albumin (New England Biolabs), 1 μl MgCl_2_ (50 mM) (Finnzymes), 3.4 μl dH_2_O, and 2 μl DNA template. All DNA extracts were diluted tenfold before they were used in PCR reactions. The setup of the touchdown PCR program consisted of the following: initial denaturation at 95 °C for 10 min followed by 5 cycles of 45 s at 95 °C, 1 min at 62 °C (reduced stepwise to 57 °C), and 45 s at 72 °C. This was followed of 40 cycles of 45 s at 72 °C, 45 s at 95 °C, 1 min at 57 °C, and 45 s at 72 °C (Perez-de-Mora et al*.* 2011). The PCR program was ended with 20 min at 72 °C. The standard used was a tenfold serial dilution of *alkB* genes from *Pseudomonas putida* GPo1, and it ranged from 10^3^ to 10^7^ copies per qPCR reaction. The standard curve was linear with a *R* ^2^ > 0.99 with a reaction efficiency of the real-time PCR of approximately 75 %.

### Data analysis

Comparison of two data sets was performed by unpaired two-tailed *t* test. Analysis of concentration correlation was done with linear regression analysis. Correlation analysis between different dependent variables was done using PAST statistics (http://folk.uio.no/ohammer/past/). All experiments were carried out with triplicate samples and with at least one independent repetition of each experiment. Data are presented as mean ± SD. *P* <0.05 is selected as cutoff for statistical significance.

## Results

### Emulsification of n-hexadecane by purified lipopeptides

**Fig. 1:**
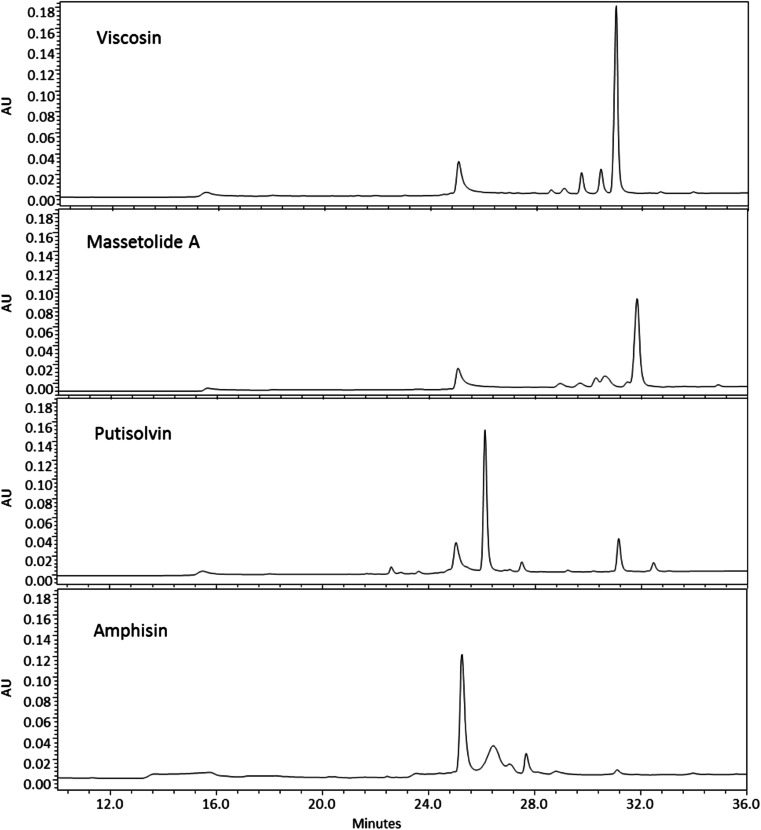
HPLC chromatograms obtained from preparations of viscosin, massetolide A, putisolvin, and amphisin purified from *P. fluorescens* SBW25, *P. fluorescens* SS101, *P. putida* 267 and *Pseudomonas* sp. DSS73, respectively. *AU* absorbance units

Lipopeptide preparations were initially analyzed by HPLC to ensure purity of compounds used for subsequent experiments. Figure 1 shows the chromatograms obtained for the lipopeptides viscosin, massetolide A, putisolvin, and amphisin. They were all characterized by dominating peaks complemented by a few minor peaks eluding slightly earlier of, or later, than the major peak. The analysis documented that pure lipopeptides were obtained as the additional peaks in the chromatograms represent derivatives of the major lipopeptides generated as a result of flexibility of the synthetases involved in lipopeptide synthesis (Stachelhaus et al. 1999) as discussed below.

**Fig. 2:**
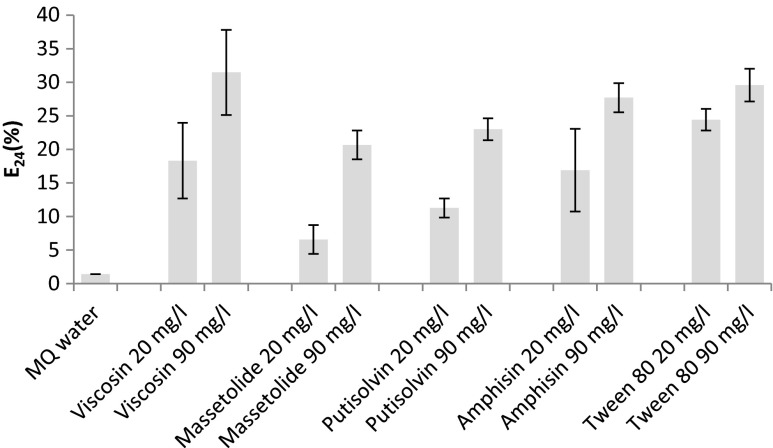
Emulsification of n-hexadecane (index E_24_ (%)) obtained for *Pseudomonas* lipopeptides viscosin, massetolide A, putisolvin, amphisin, and Tween 80. *MQ water* served as control. Values are mean of triplicates with *error bars* representing ± SD

All the *Pseudomonas* lipopeptides and the commercial synthetic surfactant Tween 80 emulsified n-hexadecane with emulsification indices of 6–24 % at 20 mg/l and 20–31 % at 90 mg/l (Fig. 2). The emulsification indexes for all compounds were dependent on the concentration, with higher values at 90 mg/l than at 20 mg/l (*P* < 0.05, except from viscosin with a *P* value of 0.055). Viscosin and amphisin had comparable emulsification powers as the synthetic surfactant Tween 80, while massetolide and putisolvin were significantly weaker emulsifiers both at 20 and 90 mg/l (*P* = 0.000451 and 0.0089 for massetolide; *P* = 0.000331 and 0.0177 for putisolvin). Viscosin was selected for subsequent studies, as it was the biosurfactant with the highest ability to emulsify n-hexadecane.

### Effect of viscosin on n-hexadecane mineralization and growth of a bacterial consortium

**Fig. 3:**
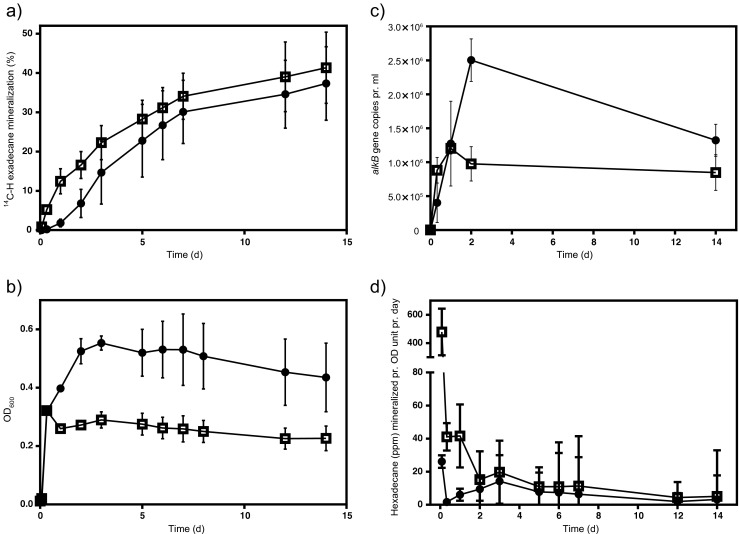
The effect of viscosin on n-hexadecane mineralization and cell growth of a diesel degrading consortium. For all panels, results for consortia treated with viscosin at 90 mg/l are symbolized by *squares*, whereas results for control consortia are shown by *circles*. **a** Hexadecane mineralization; **b** growth of the bacterial consortia measured as OD_600_; **c** the dynamics of *alkB* gene copies; **d** calculated amount of n-hexadecane mineralized per day normalized by OD_600_ units. Note that the *y*-axis in **d** has two different intervals. *Error bars* represent 1 SD with *n* = 3. Error bars not shown are smaller than symbols

The effect of viscosin on n-hexadecane mineralization and growth of the diesel-degrading bacterial consortium was studied at a concentration of 90 mg/l. The consortium treated with viscosin showed a significantly higher n-hexadecane mineralization until day 2 compared to the non-treated consortium (*P* = 0.027) (Fig. 3a). However, this stimulatory effect was only short lived, and from day 3, there was no significant difference between the viscosin-treated consortium and the control. After 14 days, the control and the viscosin-treated consortium had mineralized 37.3 % (±9.4 %) and 41.3 % (±9.1 %) n-hexadecane, respectively.

Growth of the bacterial consortium was initially assessed by OD_600_ measurements. Figure 3b shows that the control and the viscosin-treated consortia initially grew comparably and reached an OD_600_ of ca. 0.3 on day 1. Thereafter, growth of the viscosin-treated consortium was arrested while the control consortium continued to grow for three additional days and reached an OD_600_ of approximately 0.55. At the end of the experiment, growth yield (final OD_600_) of the viscosin-treated consortium was significantly lower than of the control (*P* < 0.05).

To complement OD_600_ measurements, we quantified *alkB* gene copy numbers to determine the population dynamics of bacteria harboring an alkane degradation potential in the consortia. A comparable initial increase in abundance of *alkB* genes was observed for both consortia during the first day (Fig. 3c), while the amount of *alkB* genes was significantly higher in the control than in the viscosin-treated consortium after 2 days (*P* = 0.0028). In the viscosin-treated consortia, the *alkB* gene numbers remained constant, while those in the controls decreased after day 2 so that no significant difference was observed after 14 days (*P* = 0.08). Hence, the abundance of the *alkB* genes showed the same dynamics as the growth pattern observed by OD_600_ measurements for both the control and the viscosin-treated consortia.

To assess the ability of viscosin to stimulate n-hexadecane mineralization within the first 2 days in more detail, the mineralization per time unit normalized by the bacterial biomass expressed as OD_600_ is accentuated in Fig. 3d. The viscosin-treated consortium degraded significantly more (*P* < 0.05) n-hexadecane per biomass unit than the control in the early stage of the experiment. During the remaining time of the experiment, the treated and the non-treated consortium mineralized n-hexadecane at comparable rates.

In conclusion, viscosin has a positive effect on n-hexadecane mineralization in the initial phase of the experiment while overall growth is negatively affected by the biosurfactant. Furthermore, assessment of bacterial growth by measurements of OD_600_ and *alkB* copy numbers provides comparable estimates of viscosin impact on growth dynamics.

### Degradation of viscosin

**Fig. 4:**
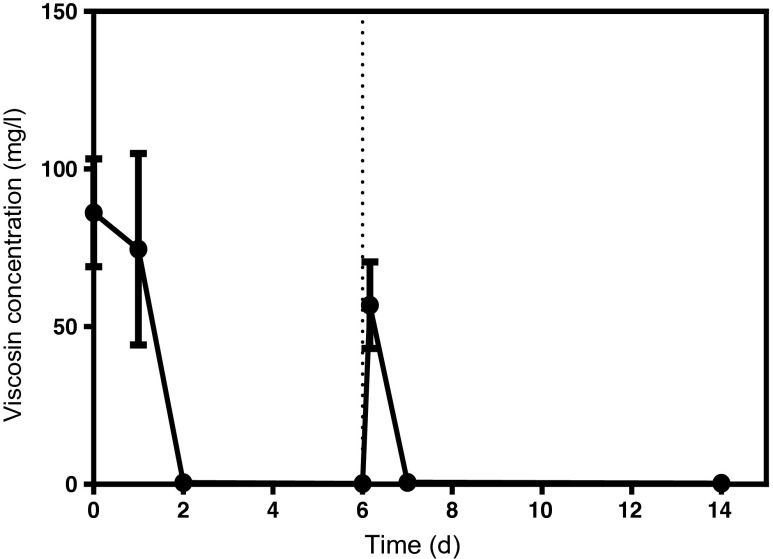
The concentration of viscosin during experiments as quantified by HPLC. *Dotted line* represents time of spiking with 60 mg/l of viscosin. *Error bars* represent 1 SD with *n* = 3. Error bars not shown are smaller than the symbols

The short-lived stimulation of n-hexadecane mineralization exerted by viscosin led us to investigate the stability of the biosurfactant in the experiment. Figure 4 shows the concentration of viscosin over 14 days as quantified by HPLC. The initial amount (90 mg/l) of viscosin could not be detected after the first 48 h. At day 6, samples were re-spiked with 60 mg/l of viscosin. The viscosin added on day 6 was not detected on day 7. Hence, we conclude that viscosin is rapidly degraded by members of the bacterial consortium.

## Discussion

### Emulsification of n-hexadecane

Viscosin, massetolide A, amphisin, and putisolvin represent different groups of *Pseudomonas* lipopeptides. The viscosin-group compounds viscosin and massetolide A both consist of nine amino acids, while members of the amphisin group have 11 amino acids in their peptide part. Finally, putisolvin represent lipopeptides with 12 amino acids and a cyclization that differs from that of the viscosin- and amphisin-group compounds (Raaijmakers et al. 2010). All four lipopeptides are powerful biosurfactants able to lower the surface tension of water to about 30 mN/m (Saini et al. 2008; de Souza et al. 2003; Kruijt et al. 2009). These compounds are synthetized by non-ribosomal peptide synthetases, which display substrate flexibility so that an individual strain produces a major compound plus a number of structural analogues in minor amounts (Raaijmakers et al. 2010). This is reflected in the current chromatograms of purified viscosin, massetolide A, putisolvin, and amphisin preparations, which resemble profiles obtained by previous researchers for viscosin, massetolide A, and putisolvin. In contrast, chromatograms of amphisin have not previously been published (Kuiper et al. 2004; Tran et al. 2007; De Bruijn et al. 2007; de Bruijn and Raaijmarkers 2009; Janek et al. 2010).

We show that all the investigated *Pseudomonas* lipopeptides emulsify n-hexadecane in a concentration-dependent manner. Few other studies have tested the emulsification activity of lipopeptide biosurfactants at the low concentrations used in the current study. Saini et al. (2008) showed that viscosin can solubilize Pennz motor oil efficiently at 7.5 mg/l, while the *Bacillus* lipopeptide surfactin yields an emulsification index of 10–20 % when tested at 10–20 mg/l against diesel oil (Whang et al. 2009), i.e., values comparable to those obtained here for *Pseudomonas* lipopeptides against n-hexadecane. Surfactin as well as pseudofactins purified from *Pseudomonas* have been analyzed for their emulsification properties at the much higher concentrations of 0.5 % (*w*/*v*) and 1,120 mg/l, respectively (Abdel-Mawgoud et al. 2008; Janek et al. 2010), where emulsification indices on n-hexadecane of approximately 65 % were reported.

Hence, the emulsification properties of the *Pseudomonas* lipopeptides and of surfactin seem to be overall comparable. However, our observations indicate that members of different *Pseudomonas* lipopeptide groups (e.g., massetolide A vs amphisin) can perform differently in n-hexadecane mineralization. Interestingly, the same holds true for structurally much related compounds within the same group of biosurfactants as massetolide A has significantly poorer emulsification properties than viscosin. These two lipopeptides only differ by a single amino acid substitution (Val vs Ile). The result underlines the importance of small structural differences for lipopeptide function, as has previously been demonstrated for their antimicrobial activities (Gerard et al. 1997). It is also worth to highlight that in studies of hexadecane emulsification properties of lipopeptides, most of them are able emulsify hexadecane to the same extent as the synthetic surfactants Tween 20, Tween 80, and Triton X-100.

### Effect of viscosin on n-hexadecane mineralization and consortium growth

We investigated the impact of viscosin on n-hexadecane mineralization and on consortium growth at a concentration of 90 mg/l, which is approximately 1.5 times the CMC of 54 mg/l (Saini et al. 2008). This concentration was selected to enable biosurfactant-mediated substrate transfer (Bouchez-Naitali et al. 1999). Further, 90 mg/l of the related lipopeptide surfactin has previously observed in situ in a natural environment harboring introduced *Bacilli* (Youssef et al. 2007).

We found that viscosin stimulated n-hexadecane mineralization during the initial 48 h of the experiment. Further, the biosurfactant inhibited growth, measured as turbidity as well as *alkB* gene copy numbers, from 24 h and onwards. Consequently, n-hexadecane mineralization per time unit normalized by the bacterial biomass was dramatically increased by viscosin amendment in the initial phase of the experiment. There are a limited number of reports on the impact of lipopeptide biosurfactants on aliphatic hydrocarbon biodegradation by bacterial consortia. Hence, Moran et al. (2000) observed that surfactin added at 80 mg/l, but not at 10 mg/l, stimulated the biodegradation, as well as cell growth by an indigenous microbial community from ship bilge waste maintained as a liquid culture. In a comparable study, Whang et al. (2008) demonstrated that surfactin at 40 mg/l stimulated diesel biodegradation as well as biomass growth by an enriched diesel-degrading bacterial consortium in batch diesel/water cultures. However, higher concentrations of surfactin inhibited degradation of petroleum hydrocarbons in the diesel/water systems prompting the authors to conclude that the possible inhibitory effects of surfactin on bioremediation needs careful determination (Whang et al. 2008).

As addition of biosurfactants may have both stimulatory and inhibitory effects on biodegradation of hydrocarbons (Moran et al. 2000; Shin et al. 2005), it is important to have in mind that a biosurfactant affects bacteria differently, depending on their individual uptake strategies of hydrophobic compounds (Bouchez-Naitali et al. 1999). Treatment of a consortium with viscosin at concentrations above the CMC would be predicted to stimulate bacteria using biosurfactant facilitated transfer, and we speculate that n-hexadecane mineralization by this fraction of the consortium was stimulated in the current study, see Owsianiak et al. (2009). Viscosin, as well as surfactin, are powerful biosurfactants but concomitantly potent antibiotics (Raaijmakers et al. 2010). Neither viscosin nor surfactin have been tested systematically for their antimicrobial activity against other bacteria (Raaijmakers et al. 2010). However, it is known that viscosin is particularly active against mycobacteria with minimal inhibitory concentrations around 10–20 mg/l (Nybroe and Sørensen 2004), concentrations which the bacteria encounter in the current experiments. *Mycobacterium* and related bacteria with hydrophobic cell walls such as *Rhodococcus* are among known alkane degraders relying on the direct access strategy (Bouchez-Naitali et al. 1999). Hence, this bacterial group might be negatively affected by viscosin in the current experiments.

### Degradation of viscosin

The short-lived stimulation of n-hexadecane mineralization by viscosin addition could be due to rapid degradation of viscosin by bacteria in the consortium. In spite of its rapid degradation, viscosin irreversibly inhibits the overall growth of the consortium, which could point toward an inhibitory role of a viscosin degradation product. A single report indicates that lipopeptides as arthrofactin and surfactin can be used as substrates by several bacteria in pure culture, and lipopeptide degradation is even carried out by mixed bacterial cultures introduced into soil microcosms (Lima et al. 2011). For more natural systems, the lipopeptides viscosinamide, tensin, and amphisin have been shown to reach 50 % degradation with 5–10 days in soil (Nielsen and Sørensen 2003). Concerning degradation products, a surfactin hydrolase has recently been identified in a *Streptomyces* strain (Hoefler et al. 2012), but so far, lipopeptide degradation products and their specific functions have, to the best of our knowledge, not been characterized in the literature.

In conclusion, the current results show that *Pseudomonas* lipopeptides are efficient emulsifiers of n-hexadecane and that a selected compound—viscosin—stimulates n-hexadecane mineralization by a diesel-degrading consortium in the initial state of the degradation process. However, the toxicity and biodegradability of viscosin appears to limit its future potential in bioremediation. Previous pure-culture studies involving other biosurfactants suggest that members of the consortium with different substrate uptake strategies might respond differently to viscosin. Further investigations employing simple consortia, including strains representing the different strategies, might unravel the complex interactions between lipopeptides and different consortium members.
